# Senescent characteristics of human corneal endothelial cells upon ultraviolet-A exposure

**DOI:** 10.18632/aging.205761

**Published:** 2024-04-26

**Authors:** Kohsaku Numa, Sandip Kumar Patel, Zhixin A. Zhang, Jordan B. Burton, Akifumi Matsumoto, Jun-Wei B. Hughes, Chie Sotozono, Birgit Schilling, Pierre-Yves Desprez, Judith Campisi, Koji Kitazawa

**Affiliations:** 1 Buck Institute for Research on Aging, Novato, CA 94945, USA; 2 Kyoto Prefectural University of Medicine, Department of Ophthalmology, Kyoto 6020841, Japan; 3 MRC Toxicology Unit, University of Cambridge, Cambridge CB2 1QR, UK; 4 California Pacific Medical Center, Research Institute, San Francisco, CA 94107, USA

**Keywords:** cellular senescence, senescence-associated secretory phenotype, RNA-Seq, proteomics, gene ontology analysis

## Abstract

Purpose: The objective of this study was to investigate the senescent phenotypes of human corneal endothelial cells (hCEnCs) upon treatment with ultraviolet (UV)-A.

Methods: We assessed cell morphology, senescence-associated β-galactosidase (SA-β-gal) activity, cell proliferation and expression of senescence markers (*p16* and *p21*) in hCEnCs exposed to UV-A radiation, and senescent hCEnCs induced by ionizing radiation (IR) were used as positive controls. We performed RNA sequencing and proteomics analyses to compare gene and protein expression profiles between UV-A- and IR-induced senescent hCEnCs, and we also compared the results to non-senescent hCEnCs.

Results: Cells exposed to 5 J/cm2 of UV-A or to IR exhibited typical senescent phenotypes, including enlargement, increased SA-β-gal activity, decreased cell proliferation and elevated expression of *p16* and *p21*. RNA-Seq analysis revealed that 83.9% of the genes significantly upregulated and 82.6% of the genes significantly downregulated in UV-A-induced senescent hCEnCs overlapped with the genes regulated in IR-induced senescent hCEnCs. Proteomics also revealed that 93.8% of the proteins significantly upregulated in UV-A-induced senescent hCEnCs overlapped with those induced by IR. In proteomics analyses, senescent hCEnCs induced by UV-A exhibited elevated expression levels of several factors part of the senescence-associated secretory phenotype.

Conclusions: In this study, where senescence was induced by UV-A, a more physiological stress for hCEnCs compared to IR, we determined that UV-A modulated the expression of many genes and proteins typically altered upon IR treatment, a more conventional method of senescence induction, even though UV-A also modulated specific pathways unrelated to IR.

## INTRODUCTION

Numerous ocular diseases have been closely associated with aging, including cataracts, dry eyes, glaucoma, and age-related macular degeneration [1, 2]. We previously investigated age-related changes in the ocular surface in both humans and mice [1, 3, 4]. The cornea, which constitutes the ocular surface, is a transparent tissue that refracts incoming light and sends it to the retina. Its transparency is maintained by a single layer of cells called corneal endothelial cells (CEnCs) that cover the posterior surface. CEnCs have poor proliferative capacity *in vivo*, as they are thought to be arrested in G1 phase [5, 6]. Pathological cell loss of the corneal endothelium results in corneal endothelial dysfunction, and leads to visual impairment and potential blindness [5].

Treatment of CEnC dysfunction includes corneal endothelial transplantation using donor corneas, which is the standard treatment worldwide, and cell injection therapy using cultured human CEnCs, which we have recently developed [7, 8]. However, pathological CEnC loss persists even after successful corneal transplantation or cell injection therapy [8, 9], leading to graft failure. Therefore, the mechanisms involved in CEnC loss need to be elucidated.

Some reports suggest a link between corneal endothelial disease and cellular senescence. A high percentage of senescence-like CEnCs in donor corneas can reduce the graft survival rate after corneal transplantation [10]. Senescence-like is defined as lacking all the characteristics of cellular senescence, but as exhibiting some important aspects. It has also been reported that CEnCs from patients with Fuchs endothelial corneal dystrophy (FECD), which is a leading cause of CEnC dysfunction in the world [11], have predominantly higher expression of p16 and p21 compared to healthy subjects [12, 13], suggesting there is a link between FECD and cellular senescence [14]. Despite this suggestion of the presence of senescent cells in the corneal endothelium, there are few reports characterizing their gene expression and phenotype.

The accumulation of senescent cells not only contributes to various etiologies associated with aging [15, 16], but also aggravates the pathological condition of diseases [3]. Recent research on cellular senescence has revealed that senescent cells acquire an inflammatory phenotype called the senescence-associated secretory phenotype (SASP) [17, 18], which changes the surrounding microenvironment over time [19, 20]. Efforts have been made to understand the phenotype of senescent cells, and cellular senescence models have been established, not only through replicative senescence [21], but also by inducing cellular senescence responses through oncogene overexpression [22], DNA damage caused by X-ray [23] or chemotherapy compounds such as doxorubicin [24].

In this study, we established a cell culture model to examine the phenotype of hCEnCs induced to senesce upon ultraviolet (UV)-A, which is one of the stress factors contributing to the pathogenesis of FECD [14]. We determined the characteristics of UV-A-induced senescent hCEnCs compared to ionizing radiation (IR)-induced senescent hCEnCs by investigating their gene and protein expression profiles.

## RESULTS

### UV-A radiation leads to a decrease in cell proliferation rate and an increase in cell size in human corneal endothelial cells

To investigate whether UV-A radiation induces senescence in corneal endothelial cells, hCEnCs were exposed to five different doses of UV-A radiation (0 J/cm^2^ as a mock, 2.5 J/cm^2^, 5 J/cm^2^, 10 J/cm^2^, 20 J/cm^2^) to determine the changes in cellular morphology using phase-contrast microscopy. As a positive control, cells induced to undergo cellular senescence upon treatment with 10 Gy IR, a DNA-damaging agent, were included [23].

**Figure 1:**
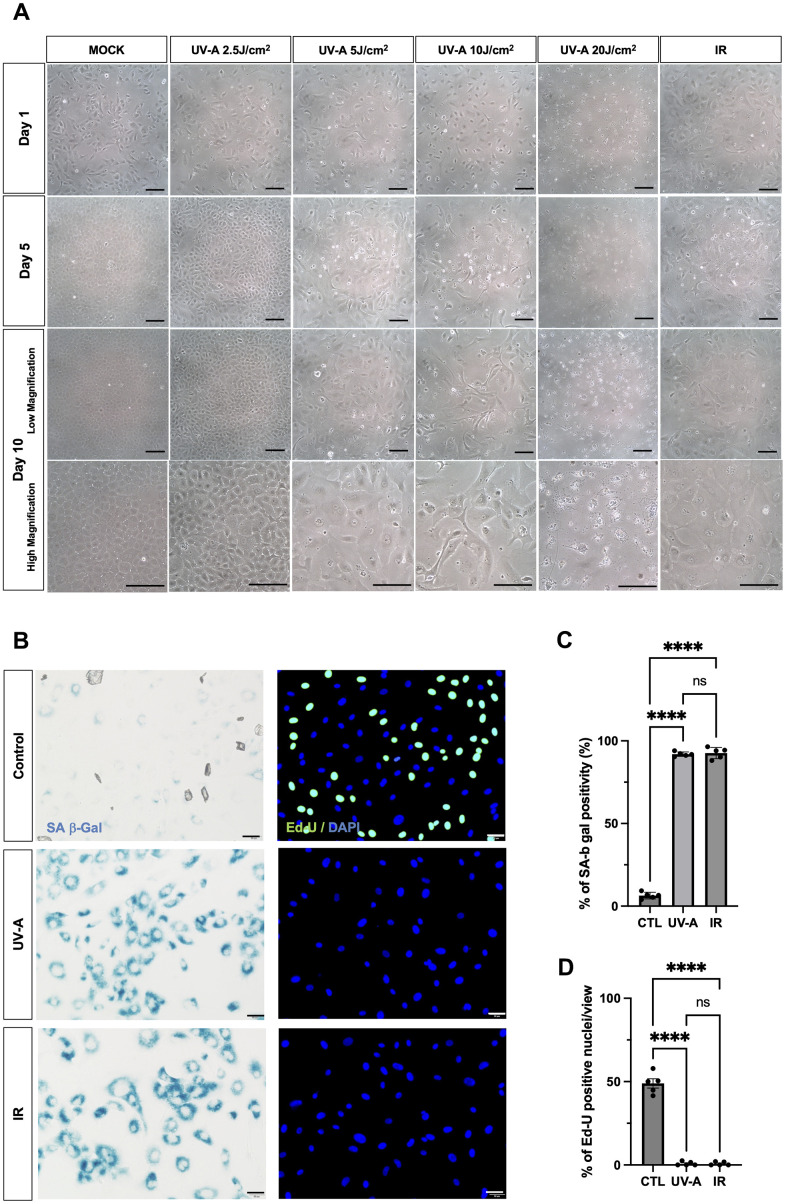
**Morphological changes of human corneal endothelial cells after UV-A exposition.** (**A**) Representative images of human corneal endothelial cells 1 (top), 5 (middle-upper) and 10 days (middle-lower: low magnification) (bottom: high magnification) after UV-A or IR treatment. Cells were either mock-treated or treated with four doses of UV-A (2.5, 5, 10, and 20 J/cm2) or IR (10 Gy). Scale bar: 100μm. (**B**) SA-β-Gal staining (left panels) and EdU labeling (right panels) of human corneal endothelial cells (hCEnCs). Cells were treated or not with 5 J/cm2 UV-A or 10 Gy IR, cultured for 10 days, and then SA-β-Gal staining and EdU labeling performed. Representative images are shown. Scale bar: 100μm. (**C**) quantification of SA-β-Gal staining, and (**D**) quantification of EdU labeling, plotted as mean of % of positive cells from five independent experiments.

The day after UV-A and IR treatment (Figure 1A, top row), cell death was observed in cells treated with 20 J/cm^2^ of UV-A radiation. On the 5th day after irradiation (Figure 1A, middle-upper row), mock-treated cells and cells exposed to 2.5 J/cm^2^ of UV-A radiation exhibited proliferation. In contrast, cells exposed to 5 J/cm^2^, 10 J/cm^2^ of UV-A radiation, or IR showed slower proliferation and increased cell size. On the 10th day after irradiation (Figure 1A, middle-lower row), cells treated with 5 J/cm^2^ and 10 J/cm^2^ of UV-A radiation, as well as IR radiation, appeared more elongated, flattened, and significantly larger than mock cells (Figure 1A, bottom row). These cells exhibited morphological features characteristic of senescent cells [25]. However, cells exposed to 10 J/cm^2^ of UV-A irradiation were unable to survive the 3 days of serum starvation, resulting in cell death. Based on these results, we then assessed the expression of senescence markers in cells exposed to 5 J/cm^2^ UV-A.

### Senescent characteristics of human corneal endothelial cells upon UV-A radiation

We irradiated hCEnCs using 5 J/cm2 UV-A or IR to characterize their senescent phenotypes. As negative controls, non-irradiated hCEnCs were subjected to serum starvation for three days. SA-β-Gal staining in UV-A- and IR-irradiated hCEnCs was detected at 91.9% and 92.6%, in UV-A- and IR-treated hCEnCs, respectively (Figure 1B, left panels), in contrast to 6.3% for control cells (Figure 1C). EdU labeling was not detectable in UV-A- and IR-irradiated hCEnCs (Figure 1B, right panels), whereas 48.9% of control cells were EdU-positive (Figure 1D).

**Figure 2:**
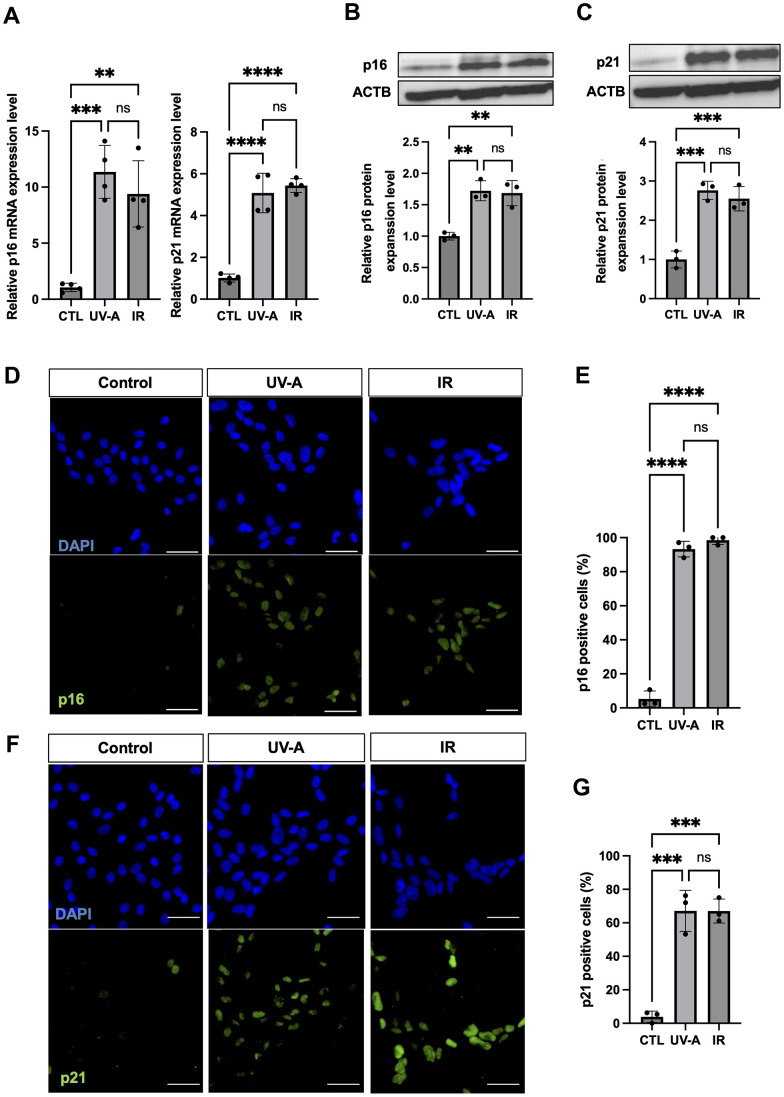
**Characteristics of senescent human corneal endothelial cells.** (**A**) RNA expression analysis by real-time PCR of p16 and p21 in control, UV-A-treated or IR-treated cells from four independent experiments. Gene expression was normalized to the housekeeping gene ACTB. B-C, Protein expression analysis by western blotting and quantification of p16 (**B**) and p21 (**C**) in control, UV-A-treated or IR-treated cells from four independent experiments. Results were plotted as mean and standard deviation from three independent experiments. Gene expression was normalized to the housekeeping protein ACTB. D-G, Immunofluorescent staining (**D**) and quantification (**E**) of p16 protein expression, and immunofluorescent staining (**F**) and quantification (**G**) of p21 protein expression, in hCEnCs treated upon UV-A or IR. Representative images are shown with scale bars indicating 100 μm. Quantification of immunofluorescent staining is presented as the mean percentage of positive cells from three independent experiments. ** = p < 0.01; *** = p < 0.001; **** = p < 0.0001; ns=not significant.

Subsequently, we assessed the expression of senescence makers p16 and p21. qRT-PCR showed significant upregulation of expression of both p16 and p21 in UV-A- and IR-treated hCEnCs compared to non-irradiated cells (Figure 2A). Western blot analysis confirmed the increase in p16 and p21 expression at the protein level in treated cells compared with controls (Figure 2B, 2C). Immunofluorescent staining for p16 revealed positive cells in 93.3% and 98.6% of UV-A- and IR-irradiated hCEnCs, respectively, compared to 5.3% in control cells (Figure 2D, 2E). Moreover, immunofluorescent staining for p21 showed positive cells in 67.1% and 67.0% of UV-A- and IR-irradiated hCEnCs, respectively, compared to 3.9% in control cells (Figure 2F, 2G).

### Investigation of the senescence response of human corneal endothelial cells using RNA-Seq analysis

**Figure 3:**
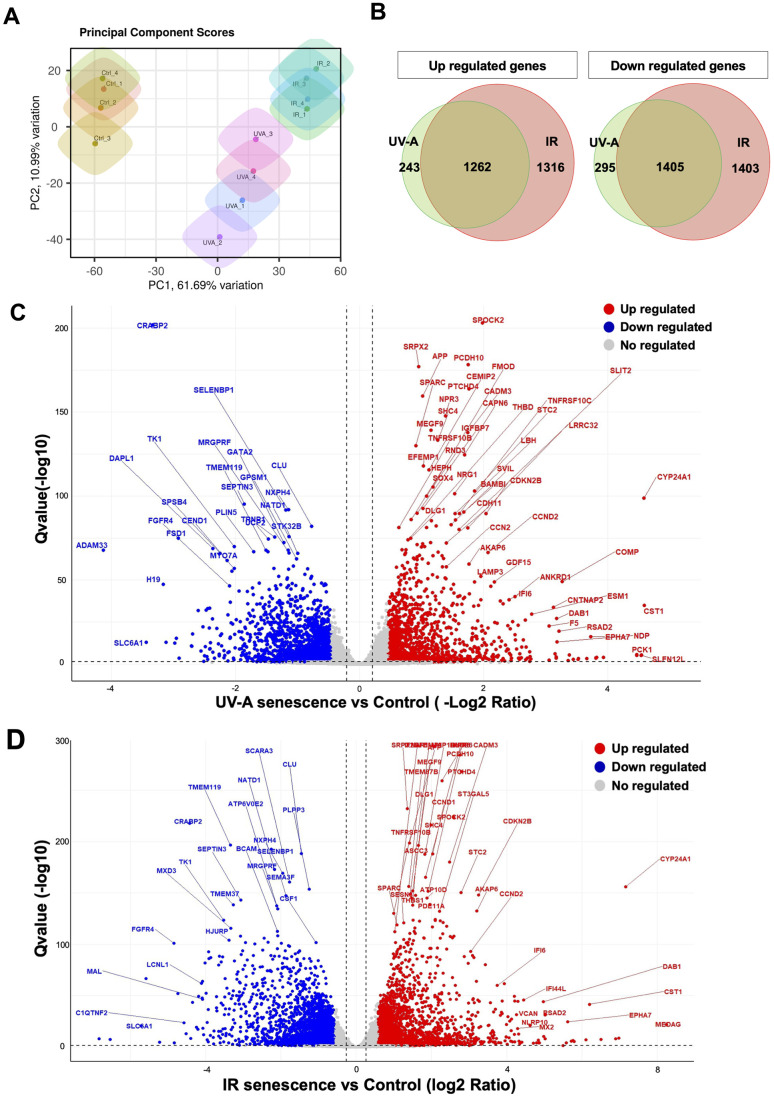
**Comprehensive transcriptional analysis of UV-A- and IR-induced senescent human corneal endothelial cells.** (**A**) Principal component score analysis of UV-A- and IR-induced senescent and non-senescent (control) hCEnCs (n=4, respectively). (**B**) Venn diagram analysis of differentially expressed genes upregulated (left) and downregulated (right) in UV-A- and IR-induced senescent hCEnCs compared to control (fold change ≥ 2, q-value ≤ 0.05). (**C**) Volcano plot comparing UV-A-induced senescent hCEnCs and control, and (**D**) Volcano plot comparing IR-induced senescent hCEnCs and control, show Q-values (−log10) vs. fold change of (log2) UV-A- or IR-induced senescent hCEnCs and control. Blue, downregulated genes; red, upregulated genes; and black, no significant change (fold change ≥ 2, q-value ≤ 0.05).

Next, we extracted total RNA from the hCEnCs treated with UV-A or IR to perform RNA-Seq analysis, aiming to explore the full spectrum of genes that are regulated upon senescence induction, in comparison to non-senescent hCEnCs used as negative controls. The total number of reads were 22366716 and 22329586, for UV-A- and IR-irradiated senescent hCEnCs, respectively, and 22198089 for the negative controls (Supplementary Figures 1, 2). Principal component analysis (PCA) was performed to identify patterns of gene expression variability across samples. The three groups, UV-A, IR, and control showed distinct patterns in PCA (Figure 3A).

A total of 3205 genes were found to be differentially expressed (DEGs), with 1505 genes upregulated and 1700 genes downregulated in UV-A-induced senescent hCEnCs compared to control cells (fold change>2 and adjusted p-value<0.05). A total of 5386 genes were found to be differentially expressed, with 2578 genes upregulated and 2808 genes downregulated in IR-induced senescent hCEnCs compared to control cells (fold change>2 and adjusted p-value<0.05). We determined that 243 and 1316 upregulated genes were unique to UV-A- and IR-induced senescent hCEnCs, respectively, while 1262 upregulated genes overlapped (Figure 3B, left panel). We also determined that 295 and 1403 downregulated genes were unique to UV-A- and IR-induced senescent hCEnCs, respectively, while 1405 downregulated genes overlapped (Figure 3B, right panel). Our RNA-Seq analysis revealed significant difference in gene expression between UV-A-induced senescent hCEnCs vs negative control cells (Figure 3C), and between IR-induced senescent hCEnCs vs negative control cells (Figure 3D).

### Gene ontology analysis of UV-A- and IR-induced senescent human corneal endothelial cells

**Figure 4:**
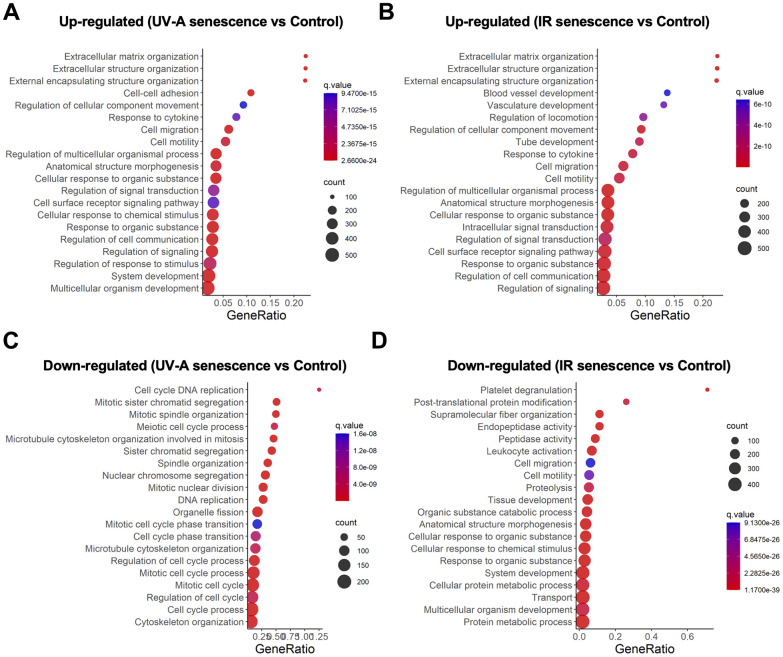
**Gene expression analysis comparing UV-A- and IR-induced senescent human corneal endothelial cells.** (**A**, **B**) Gene ontology analysis of upregulated genes comparing (**A**) UV-A-induced senescent hCEnCs and non-senescent hCEnCs (control), as well as (**B**) IR-induced senescent hCEnCs and control. (**C**, **D**) Gene ontology analysis of down-regulated genes comparing (**C**) UV-A-induced senescent hCEnCs and control, as well as (**D**) IR-induced senescent hCEnCs and control.

We then conducted gene ontology analysis and identified several significantly enriched biological processes among the DEGs. Pathway analysis revealed that the upregulated genes were enriched in pathways associated with extracellular matrix (ECM) organization, extracellular structure organization, external encapsulating structure organization, regulation of cellular component movement, response to cytokines, cell migration, and cell motility in both UV-A- and IR-induced senescent hCEnCs (Figure 4A, 4B). In contrast, there were no overlapping pathways among the top 20 downregulated pathways in cells induced to senescence by UV-A or IR (Figure 4C, 4D).

Next, we conducted a comparative analysis between UV-A- and IR-induced senescent hCEnCs. Our RNA-Seq analysis also revealed significant differences in gene expression profiles between UV-A- and IR-induced senescent hCEnCs (Supplementary Figure 2A). Pathway analysis of IR- compared to UV-A-induced senescent hCEnCs indicated that the upregulated genes were enriched in pathways associated with mitotic spindle organization, sister chromatid segregation, and spindle organization (Supplementary Figure 2B), while the downregulated genes were enriched in pathways related to defense responses to symbionts, defense responses to viruses, and responses to viruses (Supplementary Figure 2C).

### Expression of cellular senescence-, SASP-, ECM- and EMT-related genes

**Figure 5:**
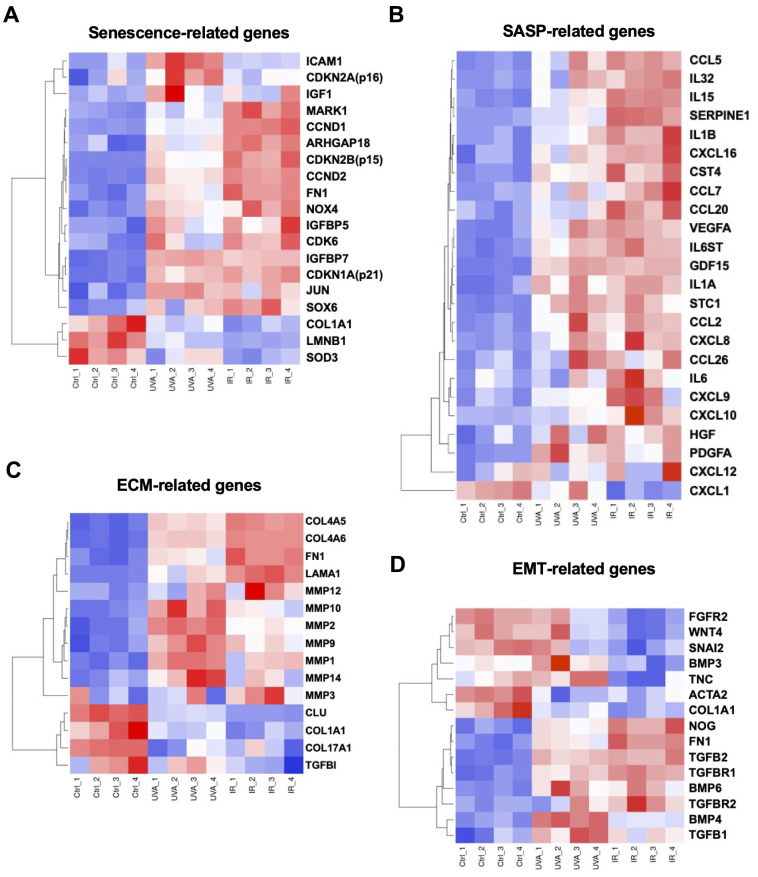
**Heatmaps of four major types of genes.** (**A**) Senescence-, (**B**) SASP-, (**C**) ECM-, and (**D**) EMT-related DEGs were picked and normalized by Z-score. Red: up-regulated expression. Blue: down-regulated expression.

It has been suggested that excessive ECM deposition, due to upregulation of epithelial to mesenchymal transition (EMT)-induced genes, was involved in the pathogenesis of FECD [14]. In our study, we focused on cellular senescence-, SASP-, ECM-, and EMT-related gene signatures. First, we selected 19 DEGs associated with cellular senescence based on the findings by Matthaei et al. [25] and Cui et al. [26]. Differential expression was observed between the different groups: CDKN2A, ICAM1, and IGF1 were more upregulated in UV-A-induced senescent cells, while CDKN2B, CCND1, CCND2, NOX4, and CDK6 were more upregulated in IR-induced senescent cells. Conversely, LMNB1 and the antioxidant SOD3 were downregulated in both UV-A- and IR-induced senescent cells (Figure 5A). We also investigated SASP-related genes, and we found that genes such as interleukins including IL1A, IL1B, IL6, IL15, and IL32 were upregulated, as well as inflammatory chemokines including CCL2, CCL5, CCL7, CCL20, CCL26, CXCL8, CXCL10, CXCL16, and CXCL26, in both UV-A- and IR-induced senescent cells (Figure 5B).

Additionally, ECM-related genes such as MMPs were mainly upregulated in the UV-A senescent group (Figure 5C). Conversely, COL4A5 and COL4A6 were upregulated, while COL1A1 and COL17A1 were downregulated, in both senescent groups. Finally, some EMT-related genes were strongly upregulated in both groups, particularly TGFB1, TGFB2, FN1, NOG, and BMP6 (Figure 5D).

### Characterization of the SASP components secreted by UV-A- and IR-induced senescent human corneal endothelial cells

**Figure 6:**
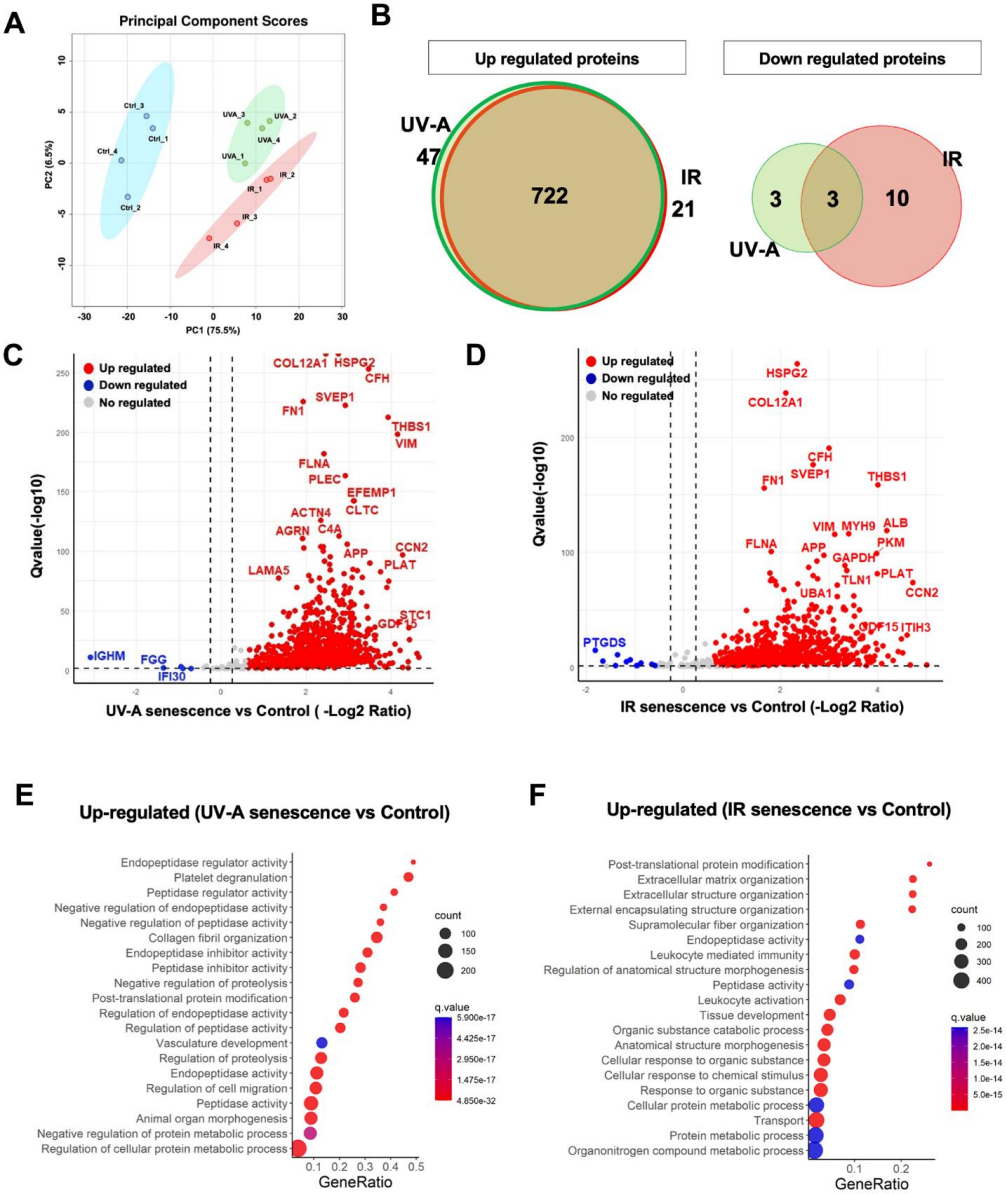
**Unbiased quantitative proteome profile of the SASP from human corneal endothelial cells.** (**A**) Conditioned media (CM) from UV-A-induced human senescent corneal endothelial cells (hCEnCs) (n = 4), IR-induced senescent hCEnCs (n = 4) and non-senescent hCEnCs (control) (n = 4) were fractionated using HPLC and analyzed using mass spectrometry. We show the principal component score analysis of CM from senescent and non-senescent hCEnCs. (**B**) Venn diagram showing unique and significantly up-regulated (left) and down-regulated (right) SASP proteins (≥ 2 unique peptides, fold change ≥ 1.5, q-value ≤ 0.05) in UV-A- and IR-induced senescent hCEnCs. (**C**, **D**) Volcano plot showing Q-values (−log10) vs. fold change of (log2) UV-A-induced senescent and control hCEnCs (**C**) as well as IR-induced senescent and control hCEnCs (**D**). Blue, downregulated proteins; red, upregulated proteins; and gray, no significant change (≥ 2 unique peptides, fold change ≥ 1.5, q-value ≤ 0.05). (**E**) Pathway and network analysis of secreted proteins that are significantly increased in the SASP from UV-A-induced senescent hCEnCs. (**F**) Pathway and network analysis of secreted proteins that are significantly increased in the SASP from IR-induced senescent hCEnCs.

The SASP is influenced by physiological states [18] and environmental stimuli [27]. Therefore, we employed a label-free, unbiased quantitative mass spectrometric approach known as data-independent acquisition (DIA) [28] to analyze the SASP factors secreted by senescent hCEnCs. The proteomics analysis was performed on conditioned media collected from UV-A- and IR-induced senescent hCEnCs as well as negative controls (non-senescent hCEnCs). This analysis identified 1,074 quantifiable protein groups with at least 2 unique peptides. Pairwise comparisons of the protein abundance for each group resulted in the identification of 766 significantly altered quantifiable proteins in the UV-A vs. control comparison, and 729 proteins in the IR vs. control comparison. PCA was conducted to identify patterns of protein expression variability across samples (Figure 6A). We found that 47 upregulated proteins were unique to UV-A-induced senescent hCEnCs compared to negative control non-senescent hCEnCs, and 21 were unique to IR-induced senescent hCEnCs, while 722 upregulated proteins were shared between both conditions (Figure 6B, left panel). Additionally, 3 downregulated proteins were unique to UV-A-induced senescent hCEnCs compared to negative control non-senescent hCEnCs, and 10 were unique to IR-induced senescent hCEnCs, with 3 downregulated proteins overlapping (Figure 6B, right panel).

Our proteomics analysis demonstrated significant differences in protein expression between UV-A-induced senescent hCEnCs and negative control cells (Figure 6C), as well as between IR-induced senescent hCEnCs and negative control cells (Figure 6D). Subsequently, we conducted gene ontology analysis and identified several biological processes significantly enriched in the differentially expressed proteins. Pathway analysis indicated that the upregulated proteins were enriched in pathways related to endopeptidase regulator activity and peptidase regulator activity in conditioned media from UV-A-induced senescent hCEnCs (Figure 6E). In contrast, pathways related to post-translational protein modification, extracellular matrix organization, and extracellular structure organization were enriched in CM from IR-induced senescent hCEnCs (Figure 6F).

Additionally, our proteomics analysis also demonstrated significant differences in protein expression between UV-A- and IR-induced senescent hCEnCs (Supplementary Figure 2D). Pathway analysis showed that the upregulated proteins were enriched in pathways related to neutrophil degranulation and neutrophil activation involved in immune responses (Supplementary Figure 2E), while the downregulated proteins were enriched in pathways associated with heterotypic cell-cell adhesion, platelet degranulation and negative regulation of the reactive oxygen species metabolic process in CM from UV-A-induced senescent hCEnCs (Supplementary Figure 2F).

### Expression of SASP proteins in UV-A-induced senescent human corneal endothelial cells

**Figure 7:**
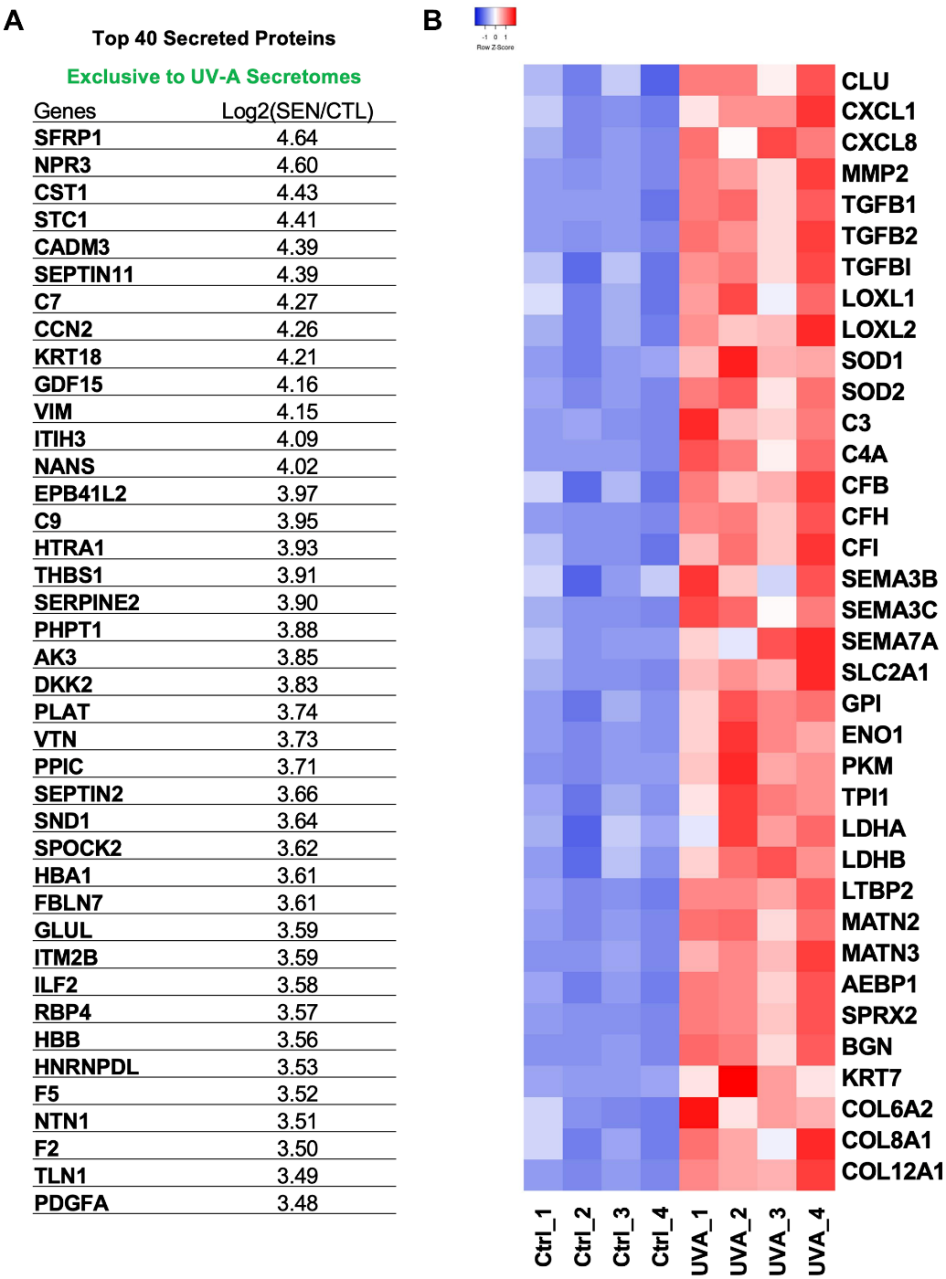
**Protein signatures of SASP factors in UV-A-induced senescent human corneal endothelial cells.** (**A**) Table of top 40 SASP proteins in UV-A induced senescence. (**B**) Heatmap of SASP proteins, which were picked and normalized by Z-score. Red: up-regulated expression. Blue: down-regulated expression.

Among the top 40 SASP proteins secreted by UV-A-induced senescent hCEnCs, key components included STC1, GDF15, C7, C9, SERPINE2, and PDGFA (Figure 7A). STC1 and GDF15 are recognized as Core SASPs, representing soluble components common to senescent cells induced by various factors [29]. To identify SASPs specific to senescent CEnCs and proteins associated with corneal endothelial diseases [30, 31], we selected and heat-mapped proteins with significantly increased secretion in UV-A-induced senescent CEnCs compared to controls (Figure 7B). We detected elevated levels of CXCL1, CXCL8, MMP2, COL6A2, COL8A1, COL12A1 and other proteins that have been previously reported as SASP factors in various cell types [29]. Furthermore, CLU, TGFBI, TGFB1, TGFB2, LOXL1, LOXL2, SEMA3, SEMA7, C3, C4A, C7, C9, CFB, CFH and CFI were also upregulated (Figure 7B). In addition, proteins associated with glycolysis, including SLC2A1, GPI, ENO1, PKM, TPI1, and LDH, were identified as significantly upregulated (Figure 7B).

## DISCUSSION

Here, we showed that cellular senescence is induced in hCEnCs upon UV-A irradiation and conducted comprehensive analyses of RNA and protein expression. We found that UV-A-induced senescent hCEnCs exhibited not only the high expression of typical cellular senescence markers, but also SASP factors which included inflammatory cytokines and chemokines previously reported in other cell types as well as factors involved in the pathogenesis of corneal endothelial disease [32, 33]. Furthermore, most of the secreted proteins significantly altered by UV-A or IR induction of senescence overlapped.

UV-A exposure elevates ROS production and triggers intracellular signaling pathways, whereas IR induces DNA damage response pathways through direct double-strand breaks [23, 34]. GO analysis of RNA-seq data revealed overlapping pathways that were up- or down-regulated in response to UV-A and IR exposure. However, in each pathway, the number of genes modulated was higher in IR-treated compared to UV-A-treated cells. Even though the RNA-seq analysis showed distinct numbers of significantly up- or down-regulated genes between IR and UV-A exposure, the difference in the number of proteins observed in the proteome was much lower. The ECM-organization pathway, which is closely associated with corneal endothelial diseases, was enriched in both UV-A- and IR-treated senescent hCEnCs using the GO analysis of RNA-seq, whereas it was only enriched in IR-treated cells using the GO analysis of proteome. Since UV-A is a more physiological stress for hCEnCs, it could represent the appropriate method for inducing cellular senescence in these cells. It may also become an alternative approach to IR, chemotherapy compounds, and hydrogen peroxide in the senescence induction of other cell types.

UV radiation is the extrinsic stress to which CEnCs are most frequently exposed. UV-A accounts for about 90% of the UV radiation reaching the earth’s surface and is known to induce ROS causing oxidative stress [34]. Oxidative stress causes molecular alternation, leading to cellular senescence [35]. Observations of UV-A intensity suggest that exposure to 5 J/cm^2^ of UV-A is roughly equivalent to one hour of noonday sun exposure during the summer [34]. Moreover, prior studies have shown that exposure to 5 J/cm^2^ of UV-A induces Nrf2-regulated antioxidant defense mechanisms in immortalized hCEnCs [36].

Previous RNA-seq results of FECD samples have reported significant changes in senescence-related genes [25, 26]. Interestingly, our results show a common change in the expression of NOX4, CDKN2A, CDKN2B, IGFBP5, FN1, and SOD3. As many reports have shown [9], the number of residual transplanted CEnCs decreases continuously, even after successful corneal transplantation, and becomes larger in size, suggesting the transition to senescence, potentially associated with a SASP that could negatively affect the surrounding microenvironment of the anterior chamber.

Among the proteins upregulated in our study, STC1 and GDF15, which are core SASP factors [29], may be important for the senescent phenotype of CEnCs. Based on previous reports, CXCL1, CXCL8, MMP2, PDGFA, TGFB1, TGFB2, LOXL1, and LOXL2 were found to be associated with corneal endothelial diseases, suggesting the presence of senescent cells [32, 33, 37–40]. Previous proteomic analysis in hereditary FECD samples showed an upregulation of canonical glycolysis and several complement factors, compared to healthy controls [30]. Our results also show an upregulation of proteins related to complement factors and canonical glycolysis, and SASPs in UV-A-induced senescent hCEnCs overlap with aqueous humor proteins in FECD patients [30]. Shotgun proteomics also identified 32 proteins, including 11 proteins related to ECM pathway, which are significantly upregulated in the Descemet’s membrane constituents of FECD patients compared to healthy controls [31]. Amongst the 11 proteins related to ECM pathway, 6 proteins, BGN, COL6A2, COL8A1, LTBP2, MATN2, MATN3, were upregulated in UV-A-induced senescent cells. These results suggest a strong association between the pathogenesis of FECD and cellular senescence.

A limitation of our study is that senescent cells are heterogeneous, even in culture. Moreover, we only studied the SASP acquired by UV-A-induced senescent hCEnCs. Another senescence induction method, such as UV-B, could trigger different SASP profiles [29]. Different approaches could help improve our knowledge of the characteristics of senescent hCEnCs. In summary, our study identified the senescent characteristics of hCEnCs upon UV-A exposure, and these findings may help to advance our understanding of the pathogenesis of corneal endothelial disease and may lead to the discovery of new therapies targeting senescent cells.

## MATERIALS AND METHODS

### Cell culture and treatments

We utilized primary CEnCs isolated from human donor corneas (CorneaGen, Seattle Eye Bank, WA, USA). The hCEnCs were cultured following established protocols with slight modifications [32, 41]. Briefly, the Descemet’s membranes with the CEnCs were stripped from donor corneas and digested at 37°C using 1 mg/mL collagenase A (Roche Applied Science, Penzberg, Germany) for 12 h. Subsequently, the hCEnCs obtained from a single donor cornea were collected following two washes with OptiMEM-I (Thermo Fisher Scientific, Inc., Waltham, MA, USA) and then seeded in one well of a 6-well plate that had been pre-coated with laminin E8 fragments (iMatrix-511; Nippi, Incorporated, Tokyo, Japan) [42].

hCEnCs were cultured at 37°C with 95% humidity and 5% CO2 in complete hCEnC medium, which consisted of OptiMEM-I (Thermo Fisher Scientific), 8% fetal bovine serum (FBS), 20 μg/mL ascorbic acid (Sigma-Aldrich, St. Louis, MO, USA), 200 mg/L calcium chloride, 0.08% chondroitin sulfate (Sigma-Aldrich), Rho-kinase inhibitor Y-27632 (10 μM) (Selleck Chemicals, Houston, TX), and penicillin-streptomycin (50 IU/ml) (Nacalai Tesque, Kyoto, Japan). Cultured hCEnCs were passaged after harvest with 10xTrypLE Select (Thermo Fisher Scientific, Inc.) treatment at 37°C for 12 min when they reached confluence and used for subsequent experiments. hCEnCs at passage 2 were used for all experiments.

### UV-A and IR treatment of hCEnCs in culture

Two 19.5-inch UV-A tubes (XX-15L; Analytik Jena US LLC), emitting 365 nm light with an irradiance of 6.5 mW/cm^2^, were employed to irradiate hCEnCs cells in 12-well cell culture plates filled with complete medium. The delivered fluence levels were 2.5, 5, 10, and 20 J/cm^2^, corresponding to irradiation times of 6.5, 13, 26, and 52 min, respectively, at 10 cm from the light source. As positive controls, senescent hCEnCs were also generated from primary hCEnCs using IR (10 Gy), as previously described [3, 4]. These cells were cultured for a period of 10 days to allow the development of a complete senescent phenotype. Both UV-A- and IR-irradiated cells were subsequently cultured in complete medium for 10 days and then subjected to a serum starvation period lasting 3 days before being analyzed. Mock-treated cells served as negative controls.

### SA-β-gal staining and EdU labeling

Cells were fixed and processed for SA- β-gal staining as per the manufacturer’s instruction (Biovision, Waltham, MA, USA). A Nikon Eclipse E800 microscope was used for imaging, and images were quantified using Image J software. Cell proliferation was evaluated by incorporation of EdU and the Click-iT EdU Cell Proliferation Assay Kit (Invitrogen, Waltham, MA). Briefly, cells were given 10 μM EdU for 24 h before fixation, permeabilized and incubated with Click-iT reaction cocktail as per the manufacturer’s instruction. A microscope (Nikon Eclipse E800) was used for imaging, and images were quantified using Image J software. >100 cells from 5 different fields were quantified per condition, and all experiments were done in triplicate.

### qRT-PCR analysis

RNA was isolated from cultured cells and homogenized tissues using the Bioline Isolate II RNA Mini Kit (Meridian Bioscience, Cincinnati, OH, USA) as recommended by the supplier. Complementary DNA was synthesized from 500-1000 ng of total RNA using High-Capacity cDNA Reverse Transcription Kit (Thermo Fisher Scientific). qRT-PCR was performed using the LightCycler® 480 Real-Time PCR System (Roche Applied Science, Penzberg, Germany) as described previously [43]. The 2 ×−ΔΔCt method was used to calculate expression levels normalized to human or mouse ACTB Endogenous Control (Thermo Fisher Scientific). All the primers used for the experiments are listed in Supplementary Table 1.

### Western blotting

Cells were washed with ice-cold PBS and lysed with RIPA buffer supplemented with 2-mercaptoethanol (final concentration 6%) and Halt Protease and Phosphatase Inhibitor Cocktail (Thermo Fisher Scientific), then boiled for 5min at 95°C. Equal amounts (10-30 μg) of samples were loaded and proteins were separated by SDS-PAGE using 4-12 % Bis-Tris gels (Bio-Rad, Hercules, CA, USA), followed by transfer to PVD membranes using iBlot Dry Blotting Gel Transfer System (Thermo Fisher Scientific). Membranes were blocked for 1 h in 5% BSA-TBST at room temperature, and then incubated with primary antibodies overnight at 4°C. Blots were washed and incubated with HRP-conjugated secondary antibodies for 1 hour at room temperature, and detection was performed using enhanced chemiluminescence. Primary antibodies were against p16 (Abcam, #ab108349, 1:500 dilution) and p21 (Cell Signaling Technology, #2947s, 1:1000 dilution). Secondary antibodies were HRP-conjugated goat anti-rabbit (Bio-Rad) and goat anti-mouse (Bio-Rad) antibodies. An antibody against beta actin (Sigma, #A2228, 1:10,000 dilution) was used for loading control.

### Immunofluorescence staining

Cells in an 8-well chamber glass slide (Thermo Fisher Scientific) were fixed with 4% paraformaldehyde at 4°C for 15 min. After PBS wash, the samples were permeabilized with 0.3% Triton-X-100 at room temperature for 15 min. The samples were then blocked with 1% bovine serum albumin (Jackson ImmunoResearch Inc.) and incubated overnight with the p16 (Abcam, #ab108349, 1:500 dilution) or p21 (Abcam, #ab109520, 1:200 dilution) primary antibody at 4°C. The samples were then washed with PBS and incubated at room temperature for 1 h with the secondary antibody (Alexa Fluor 488-labeled anti-rabbit IgG, #A-11034, Life Technologies Corp.) at a dilution of 1:1000. Finally, DAPI was added after washing, and the signal was detected using a fluorescence microscope (Nikon Eclipse E800). Images were quantified using Image J software and >100 cells from 3 different fields were quantified per condition.

### RNA-Seq analysis

We extracted RNA from UV-A- and IR-induced senescent hCEnCs, and non-senescent hCEnCs (4 samples each) to perform RNA-Seq analysis. RNA quality and quantity were determined, then RNA samples were sent to BGI Americas Corporation for sequencing on DNBSEQ platform. In parallel, RNA was reverse transcribed and qRT-PCR reactions were performed for p16 and p21. To evaluate the quality of the raw RNA-seq data, we initially performed a quality assessment using FastQC (v0.12.1; https://www.bioinformatics.babraham.ac.uk/projects/fastqc/). To remove adapter sequences and low-quality reads, we used TrimGalore (v4.3; https://www.bioinformatics.babraham.ac.uk/projects/trim_galore/). The resulting trimmed reads were aligned to the reference genome (GRCh38) using HISAT2 (v2.2.1) [44]. Subsequently, we converted the output SAM files to sorted BAM files using SAMtools (v1.12) [45] for downstream analysis. Mapped reads were assigned to their corresponding genes using FeatureCounts (v2.0.3) [46], which generated count data for each gene. The raw read counts were then normalized using DESeq2 (v1.8.3) [47]. For exploratory analysis, differential gene expression analysis, and enrichment analysis, we utilized iDEP [48]. Differential gene expression analysis was performed using DESeq2 (v1.8.3) in R (v4.2.3; https://www.R-project.org/) and RStudio (v2023.03.0+386; https://www.rstudio.com/). Gene ontology (GO) and pathway analysis for the identified differentially expressed genes (DEGs) were conducted using the clusterProfiler (v4.6.2) package [49].

### Conditioned media

Irradiated or non-senescent mock hCEnCs were cultured in complete media, washed three times with PBS and placed in OptiMEM-I (serum- and supplement-free media) (Thermo Fisher Scientific) for 2 days. Subsequently, cells were washed with PBS and placed in OptiMEM-I, and conditioned media (CM) were collected after 24 h. Collected CM were used for mass spectrometry analysis.

### Isolation of hCEnCs SASP proteins

A detailed list of reagents and resources used in the proteomics analysis, including vendors and catalog numbers, is available in the Reagent and Resource Table (Supplementary Table 2). Briefly, protein sample processing was performed as follows: CM from senescent hCEnCs (UV-A-treated, IR-treated, and quiescent control cells [n = 4 each]) were collected as previously described [29]. Salt and other media components were removed using 3 kDa cutoff columns (Amicon Centrifugal Filters), and SASP protein extracts were subsequently lysed using lysis buffer (5% SDS and 50 mM TEAB). Each extract was reduced by incubation with 20 mM dithiothreitol in 50 mM TEAB for 10 min at 50°C, and subsequently alkylated with 40 mM iodoacetamide in 50 mM TEAB for 30 min at RT in the dark. Extracts were acidified to yield pH < 1 using phosphoric acid (v/v) and subsequently 100 mM TEAB in 90% methanol was added.

The entire protein extracts were spun through micro S-Trap columns (Protifi). Subsequently, the S-Trap columns were washed with 90% methanol in 100 mM TEAB, placed in clean elution tubes and incubated with trypsin digestion buffer (50 mM TEAB, pH ~8) at a 1:25 ratio (protease:protein, wt:wt) overnight. Peptides were then sequentially eluted with 50 mM TEAB and 0.5% formic acid, and 50% acetonitrile in 0.5% formic acid. Both fractions were pooled together, vacuum dried and re-suspended in 0.2% formic acid for desalting. The desalted peptides were concentrated and re-suspended in aqueous 0.2% formic acid for mass spectrometry-based quantitative analysis.

### Mass spectrometric data-independent acquisition (DIA)

Eight microliters of each sample were diluted with 2% acetonitrile (ACN) in 0.1% formic acid to obtain a concentration of 400 ng/μL. One microliter of indexed Retention Time Standard (iRT, Biognosys, Schlieren, Switzerland) was added to each sample as an internal standard, thus bringing up the total volume to 20 μl [50]. Reverse-phase HPLC-MS/MS data were collected with a Waters M-Class HPLC (Waters, Milford, MA, USA) coupled online to a ZenoTOF 7600 system (SCIEX, Framingham, MA) with an OptiFlow Turbo V Ion Source (SCIEX). A microelectrode with a flow rate of 1-10 μL/min was used. The solvent system consisted of 0.1% FA in water (solvent A) and 99.9% ACN, 0.1% FA in water (solvent B).

Digested peptides (400 ng) were loaded onto a Luna C_18_ micro trap column (0.3 x 20 mm, 5 μm particle size; Phenomenex, Torrance, CA, USA) over 2 min at 10 μl/min with 100% solvent A. Peptides (400 ng) were eluted on a Kinetex XB-C_18_ analytical column (150 x 0.3 mm, 2.6 μm particle size; Phenomenex) at 5 μl/min using the following gradient: linear from 5% to 32% of solvent B in 120 min, linear from 32% to 80% of solvent B in 1 min, down to 5% of solvent B in 1 min, and held at 5% of solvent B for 5 min. The total gradient length was 130 min. Each sample was acquired in DIA mode [28] [51, 52]. The following parameters were used for all acquisitions: ion source gas 1 at 10 psi, ion source gas 2 at 25 psi, curtain gas at 30 psi, CAD gas at 7 psi, source temperature at 200°C, column temperature at 30°C, polarity set to positive, and spray voltage at 5000 V. The survey MS1 spectra were acquired in the range of 395-1005 Da, with an accumulation time of 100 ms, a collision energy of 10 V with 0 V CE spread, and a declustering potential of 80 V with 0 V DP spread. All channels were enabled and the time bins to sum were set to 8. MS2 spectra were collected within the same range as the MS1 spectra using 80 variable width windows with Zeno pulsing enabled, an accumulation time of 25 ms, dynamic collision energy enabled, and charge state 2 selected. All channels were enabled and the time bins to sum were again set to 8. The total cycle time was 2.5 seconds.

### DIA data processing and statistical analysis

DIA data was processed in Spectronaut v17 (version 17.6.230428.55965; Biognosys) using directDIA. Data was searched against the *homo sapiens* proteome with 20,380 protein entries (UniProtKB-TrEMBL reviewed), accessed on 01/29/2021. Trypsin/P was set as digestion enzyme and two missed cleavages were allowed. Cysteine carbamidomethylation was set as fixed modification, and methionine oxidation and protein N-terminus acetylation as variable modifications. Data extraction parameters were set as dynamic. Identification was performed using 1% precursor and protein q-value (experiment). Quantification was based on MS2 area, local normalization was applied, and iRT profiling was selected. Differential protein expression analysis was performed using a paired t-test, and q-values were corrected for multiple testing, specifically applying group-wise testing corrections using the Storey method [48]. Protein groups with at least two unique peptides, q-value < 0.05, and absolute Log2 (fold change) > 0.58 were significantly altered and are listed in Supplementary Table 2.

### Statistics

All data with error bars are presented as mean ± S.E.M, and the individual data points (dots) are presented in the bar graphs. Statistical analyses were performed using Prism 9 software with 9.2.0 (283) (GraphPad, La Jolla, CA, USA). Comparisons between groups were conducted with one-way analysis of variance (ANOVA), followed by the post hoc Dunnett’s multiple comparison test. Welch’s adjusted t-test (also called unequal variances t-test), a modified Student’s t-test, was also used under the assumption of unequal variances. Most of the cell culture experiments were done in triplicate and reproduced at least three times independently.

### Data availability statement

Raw data sets for RNA-seq have been deposited in GEO (accession number: GSE250224). Data accession, raw data and complete MS data sets have been uploaded to the MassIVE repository of the Center for Computational Mass Spectrometry at UCSD, and can be downloaded using the following link: https://massive.ucsd.edu/ProteoSAFe/dataset.jsp?task=d524615a02754fc09d1c674f8c810ef5(MassIVE ID number: MSV000093538; ProteomeXchange ID: PDX047387).

## Supplementary Material

Supplementary Figures

Supplementary Tables
