# Supplementary material for: Senescent characteristics of human corneal endothelial cells upon ultraviolet-A exposure
Source: Aging (Albany NY). 2024 Apr 26;16(8):6673–93. doi: 10.18632/aging.205761 (PMC11087119; doi:10.18632/aging.205761)
Supplement: Supplementary Figures [file aging-16-205761-s001.pdf]

SUPPLEMENTARY FIGURES

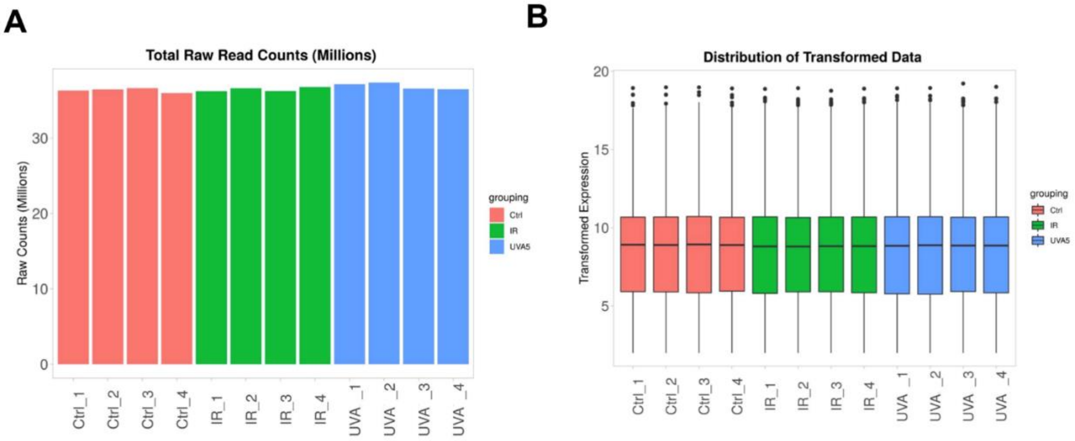

Supplementary Figure 1. RNA-Seq data profile. (**A**) Total raw read counts of all RNA-Seq samples. (**B**) Distribution of transformed data of all RNA-Seq samples.

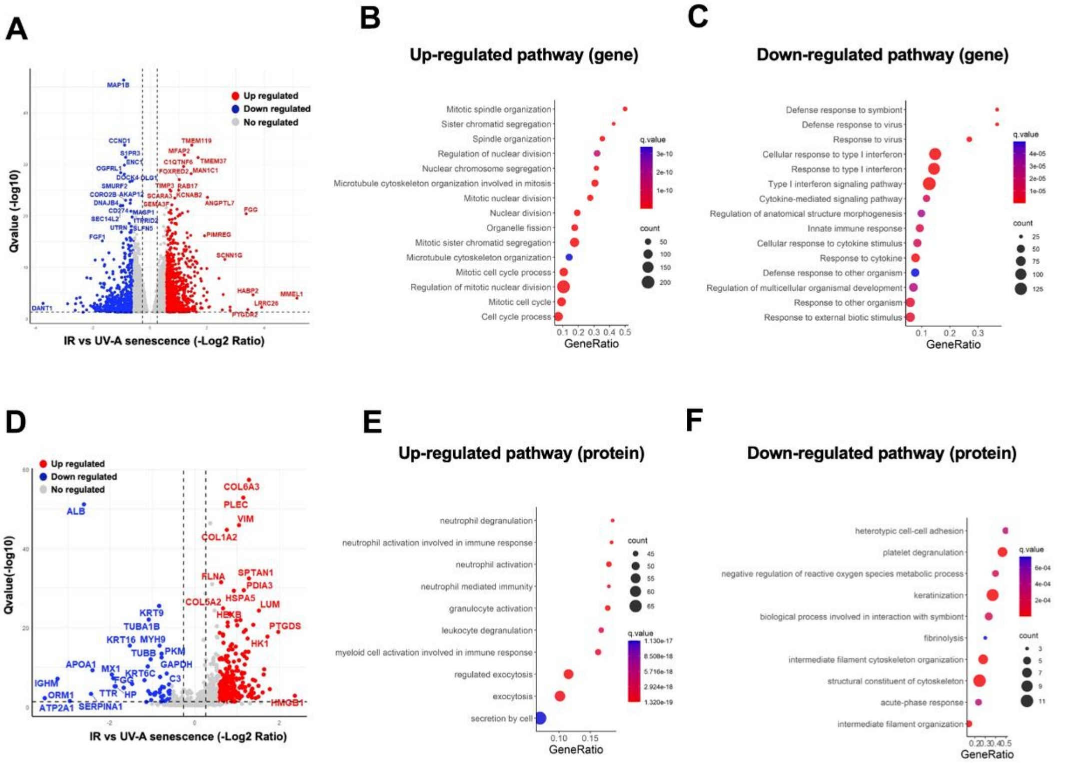

Supplementary Figure 2. Comparison of gene and protein expression patterns between UV-A- and IR-induced human senescent corneal endothelial cells. (**A**) Volcano plot comparing IR- and UV-A-induced senescent hCEnCs. Volcano plot showing Q-values (−log10) vs. fold change of (log2) IR- and UV-A-induced senescent hCEnCs. Blue, downregulated genes; red, up-regulated genes; and black, no significant change (fold change ≥ 2, q-value ≤ 0.05). (**B**, **C**) Gene ontology analysis of (**B**) up-regulated and (**C**) down-regulated genes comparing IR- and UV-A-induced senescent hCEnCs. (**D**) Volcano plot showing Q-values (−log10) vs. fold change of (log2) IR- and UV-A-induced senescent hCEnCs. Blue, downregulated proteins; red, upregulated proteins; and gray, no significant change (≥ 2 unique peptides, fold change ≥ 1.5, q-value ≤ 0.05). (**E**, **F**) Pathway and network analysis of secreted proteins that are significantly (**E**) increased and (**F**) decreased in the SASP from UV-A-induced senescent hCEnCs.
