# Supplementary material for: Senescent characteristics of human corneal endothelial cells upon ultraviolet-A exposure
Source: Aging (Albany NY). 2024 Apr 26;16(8):6673–93. doi: 10.18632/aging.205761 (PMC11087119; doi:10.18632/aging.205761)
Supplement: Supplementary Tables [file aging-16-205761-s002.pdf]

SUPPLEMENTARY TABLES

Supplementary Table 1. Assay IDs for TaqMan probes for quantitative reverse transcription PCR (qRT-PCR).

Gene symbols	Species	Assay IDs for TaqMan probes
Cdkn2a	Human	Hs02902543_mH
Cdkn1a	Human	Hs00355782_m1

Supplementary Table 2. Reagents and resources for proteomics.

Reagent or resource	Source	Identifier
Triethylammonium bicarbonate	Sigma	T7408
Concentrating Tubes	Source	Identifier
SDS	Fisher Scientific	BP166-500
BCA Protein Assay Kit	Pierce	23225
S-Trap micro spin columns	ProtiFi	N/A
Dithiothreitol (DTT)	Sigma	D9779
Iodoacetamide (IAA)	Sigma	I1149
Phosphoric Acid	Sigma-Aldrich	79622-1KG
Trypsin (sequencing grade)	Promega	V5113
Methanol	Burdick and Jackson	BJLC230-4
Water (HPLC grade)	Burdick and Jackson	AH365
Acetonitrile (HPLC grade)	Burdick and Jackson	AH015
Formic Acid	Sigma	94318
HLB Oasis SPE Cartridges	Waters	186003908
Indexed Retention Time Standard (iRT)	Biognosys	Ki-3002-2
Dionex Ultimate 3000 uHPLC	Thermo Fisher Scientific	https://www.thermofisher.com/us/en/home/industrial/chromatography/liquid-chromatography-lc/hplc-uhplc-systems/ultimate-3000-hplc-uhplc-systems.html
Orbitrap Exploris 480 mass spectrometer	Thermo Fisher Scientific	BRE725533
Acclaim PepMap 100 C_{18} pre-column	Thermo Fisher Scientific	164199
Acclaim PepMap 100 C_{18} analytical column	Thermo Fisher Scientific	164570
Spectronaut Software	Biognosys	version 15.1.210713.50606
R	R Development Core Team, 2011	https://www.r-project.org
RStudio	RStudio: Integrated Development for R. RStudio, Boston, MA	https://www.rstudio.com, Version 1.3.1093
VennDiagram (R)	Chen, 2018 (cite)	https://cran.r-project.org/web/packages/VennDiagram/index.html
ggplot2(R)	Wickham, 2016 (cite)	https://ggplot2.tidyverse.org/
Proteomics Data	This paper, MassIVE	MSV000090204
Proteomics Data	This paper, ProteomeXchange	PXD03625
